# Exercise and Attitude for Exoskeletons: The Mediation of Technology Anxiety and Perceived Usefulness

**DOI:** 10.1093/geroni/igab046.2481

**Published:** 2021-12-17

**Authors:** Seolah Lee, Do Kyung Yoon, Miseon Kang, Kwang Joon Kim, YoonMyung Kim, Yun Mook Lim, Hey Jung Jun

**Affiliations:** 1 Yonsei University, Seoul, Seoul-t'ukpyolsi, Republic of Korea; 2 Severance Hospital, Yonsei University College of Medicine, Seoul, Seoul-t'ukpyolsi, Republic of Korea

## Abstract

The present study aimed to examine the effects of regular exercise on attitude towards using exoskeletons and the mediating effects of technology anxiety and perceived usefulness among Korean older adults. Data was collected through online recruitment in February 2021. The sample comprised 310 people (Age: 65-89, M=70.18, SD=4.58) who did not report dementia. The dependent variable was the attitude towards using technology, especially lower limb exoskeleton robots for exercise. The independent variable, regular exercise, was coded as a binary variable. The mediating variables were technology anxiety and perceived usefulness measured by the sum of three questions about exoskeleton robots, respectively. Gender, age, education level, and household income were included as control variables. The mediating effect was estimated by serial path analysis and bootstrapping (model fit indices: χ2=18.400, df=8, p<0.05, RMSEA=0.065, CFI=0.973, TLI=0.940, SRMR=0.044). Results showed the total effect of regular exercise was significant (B=1.253, p<0.01) and the total indirect effect of it was significant(B=1.540, p<0.001). There was no significant direct effect of regular exercise on the attitude towards using technology. The association between regular exercise and the attitude of using technology was completely mediated by perceived usefulness (B=1.439, CI=0.569-2.358, p<.01). Although technology anxiety had no mediating effect alone, the serial mediating effect via the path from technology anxiety to perceived usefulness was significant (B=0.119, CI=0.004-0.332, p<.05). This study will be the first empirical study to examine the effects of health habits by expanding the senior technology acceptance model for older adults in Korea.

